# Analysis of the pan-Asian subgroup of patients in the NALA Trial: a randomized phase III NALA Trial comparing neratinib+capecitabine (N+C) vs lapatinib+capecitabine (L+C) in patients with HER2+metastatic breast cancer (mBC) previously treated with two or more HER2-directed regimens

**DOI:** 10.1007/s10549-021-06313-5

**Published:** 2021-09-23

**Authors:** Ming Shen Dai, Yin Hsun Feng, Shang Wen Chen, Norikazu Masuda, Thomas Yau, Shou Tung Chen, Yen Shen Lu, Yoon Sim Yap, Peter C. S. Ang, Sung Chao Chu, Ava Kwong, Keun Seok Lee, Samuel Ow, Sung Bae Kim, Johnson Lin, Hyun Cheol Chung, Roger Ngan, Victor C. Kok, Kun Ming Rau, Takafumi Sangai, Ting Ying Ng, Ling Ming Tseng, Richard Bryce, Judith Bebchuk, Mei Chieh Chen, Ming Feng Hou

**Affiliations:** 1 Department of Hematology-Oncology, Tri-Service General Hospital, Taipei, Taiwan; 2 Department of Hematology-Oncology, Chi Mei Medical Center-Yongkang Branch, Tainan, Taiwan; 3 Department of Hematology-Oncology, Chi Mei Medical Center-LiouYing Branch, Tainan, Taiwan; 4 Department of Surgery and Breast Oncology, National Hospital Organization Osaka National Hospital, Osaka, Japan; 5 Department of Medicine, The University of Hong Kong, Hong Kong, Hong Kong; 6 Department of Surgery, Changhua Christian Hospital, Changhua, Taiwan; 7 Division of Medical Oncology, Department of Oncology, National Taiwan University Hospital, Taipei, Taiwan; 8 Department of Medical Oncology, National Cancer Centre Singapore, Singapore, Singapore; 9 Department of Medical Oncology, OncoCare Cancer Centre, Singapore, Singapore; 10 Department of Hematology-Oncology, Hualien Tzu Chi Hospital, Hualien, Taiwan; 11 Department of Surgery, The University of Hong Kong Li Ka Shing Faculty of Medicine, Hong Kong, Hong Kong; 12 Center for Breast Cancer, National Cancer Center, Goyang-si, South Korea; 13 Department of Haematology and Oncology, National University Cancer Institute, Singapore, Singapore; 14 Department of Oncology, Asan Medical Center, Seoul, South Korea; 15 Department of Hematology-Oncology, Mackay Memorial Hospital, Taipei, Taiwan; 16 Department of Medical Oncology, Yonsei Cancer Center, Yonsei University College of Medicine, Seoul, South Korea; 17 Department of Clinical Oncology, Queen Elizabeth Hospital, Hong Kong, Hong Kong; 18 Division of Medical Oncology, Kuang Tien General Hospital Cancer Center, Taichung, Taiwan; 19 Department of Hematology-Oncology, E-Da Cancer Hospital, Kaohsiung, Taiwan; 20 Department of Breast and Thyroid Surgery, Kitasato University School of Medicine, Sagamihara, Kanagawa Japan; 21 Department of Clinical Oncology, Tuen Mun Hospital, Hong Kong, Hong Kong; 22 Department of Surgery, Taipei Veterans General Hospital, Taipei, Taiwan; 23 Puma Biotechnology Inc., Los Angeles, USA; 24 Department of Biostatistics, Puma Biotechnology Inc., Los Angeles, USA; 25 Clinical Development and Medical Affairs, CANbridge Pharmaceuticals Inc., Taipei, Taiwan; 26 Division of Breast Oncology and Surgery, Kaohsiung Medical University Chung-Ho Memorial Hospital, No.100, Tzyou 1st Road, Kaohsiung, 807 Taiwan

**Keywords:** Neratinib, HER2-positive breast cancer, Brain metastases, CNS metastases, Tyrosine kinase inhibitor, Lapatinib

## Abstract

**Purpose:**

Neratinib, an irreversible pan-HER tyrosine kinase inhibitor, has demonstrated systemic efficacy and intracranial activity in various stages of HER2+breast cancer. NALA was a phase III randomized trial that assessed the efficacy and safety of neratinib+capecitabine (N+C) against lapatinib+capecitabine (L+C) in HER2+ metastatic breast cancer (mBC) patients who had received ≥ 2 HER2-directed regimens. Descriptive analysis results of the Asian subgroup in the NALA study are reported herein.

**Methods:**

621 centrally assessed HER2+ mBC patients were enrolled, 202 of whom were Asian. Those with stable, asymptomatic brain metastases (BM) were eligible for study entry. Patients were randomized 1:1 to N (240 mg qd) + C (750 mg/m^2^ bid, day 1–14) with loperamide prophylaxis or to L (1250 mg qd) + C (1000 mg/m^2^ bid, day 1–14) in 21-day cycles. Co-primary endpoints were centrally assessed progression-free survival (PFS) and overall survival (OS). Secondary endpoints included time to intervention for central nervous system (CNS) disease, objective response rate, duration of response (DoR), clinical benefit rate, and safety.

**Results:**

104 and 98 Asian patients were randomly assigned to receive N+C or L+C, respectively. Median PFS of N+C and L+C was 7.0 and 5.4 months (*P* = 0.0011), respectively. Overall cumulative incidence of intervention for CNS disease was lower with N+C (27.9 versus 33.8%; *P* = 0.039). Both median OS (23.8 versus 18.7 months; *P* = 0.185) and DoR (11.1 versus 4.2 months; *P* < 0.0001) were extended with N+C, compared to L+C. The incidences of grade 3/4 treatment emergent adverse events (TEAEs) and TEAEs leading to treatment discontinuation were mostly comparable between the two arms. Diarrhea and palmar-plantar erythrodysesthesia were the most frequent TEAEs in both arms, similar to the overall population in incidence and severity.

**Conclusion:**

Consistent with the efficacy profile observed in the overall study population, Asian patients with HER2+ mBC, who had received ≥ 2 HER2-directed regimens, may also benefit from N+C. No new safety signals were noted.

**Clinical trial registration:**

NCT01808573

**Supplementary Information:**

The online version contains supplementary material available at 10.1007/s10549-021-06313-5.

## Introduction

Breast cancer has been the most prevalent cancer and the leading cause of cancer death among women. The number of patients with newly diagnosed breast cancer has surged from 1.7 million in 2012 to over 2.1 million in 2018, according to the GLOBOCAN database [1, 2]. Breast cancer alone was estimated to account for 6.6% of all cancer deaths in 2018 [3]. Of note, the increases in both incidence and breast cancer-related mortality has been exceedingly rapid and conspicuous in the patients who are from the Asian region [4]. In 2018, both the newly diagnosed cases and breast cancer deaths in Asia comprised more than 40% of the cases reported globally [1, 3]. Such a massive increase has largely been attributed to westernization of lifestyle, as well as enhanced awareness and screening [4]. Racial/ethnic disparities in breast cancer have been vastly recognized, in terms of epidemiology, tumor characteristics, genetic predisposition, and outcomes [4, 5]. In general, Asian patients tend to be younger at disease onset, present with tumors that are estrogen receptor-negative (ER−), human epidermal growth factor receptor 2-positive (HER2+), and have a higher histological grade, compared with their western counterparts [3, 6, 7]. Most importantly, these features have also been identified as important risk factors for brain metastases (BM) in breast cancer patients [8–10]. While the incidence of BM ranges between 10 and 16% in metastatic breast cancer (mBC) patients [8, 11], it may double to 22–36% among those with HER2+ tumors [12]. CNS involvement severely compromises the quality of life and prognosis of mBC patients, limiting their overall survival (OS) to 30 months [9, 13].While survival of the mBC patients may be extended with trastuzumab, development of central nervous system (CNS) diseases has been shown to be widely inevitable and occurs in around one-third of the mBC patients [8, 14]. Moreover, one retrospective study reported that progressive CNS diseases may account for half of the deaths among trastuzumab-treated mBC patients [15]. Therefore, therapeutic strategies for HER2+ mBC patients following trastuzumab failure are warranted, especially for those with BM.

Anti-HER2 tyrosine kinase inhibitors (TKIs) are a class of small-molecule drugs that have been developed to bypass trastuzumab resistance. Following the introduction of lapatinib, a reversible epidermal growth factor receptor (EGFR) and HER2 TKI, numerous irreversible inhibitors have been developed to augment HER2 inhibition. Neratinib is a potent, irreversible inhibitor of EGFR, HER2, and HER4 [16, 17]. By binding irreversibly to the cysteine residue within the adenosine triphosphate (ATP)-binding pocket of HER1, HER2, and HER4 receptors, neratinib mediates sustained inhibition of receptor phosphorylation and downstream signal transduction [18]. In preclinical studies, the selective antitumor activity of neratinib has been showcased in HER2- and EGFR-expressing and trastuzumab-resistant cell lines [16, 18, 19].

Neratinib was initially approved for the extended adjuvant treatment of patients with early-stage HER2+ breast cancer, based on the favorable results of the ExteNET study [20]. At 5-year follow-up, neratinib monotherapy was associated with improved CNS outcomes, in terms of cumulative incidence of CNS recurrences and CNS disease-free survival, in intent-to-treat (ITT) population, hormone receptor [HR] positive patients who initiated study treatment within 1 year of completing prior trastuzumab-based therapy, and patients with residual disease after neoadjuvant therapy [21]. Its activity against CNS metastases has also been demonstrated in NEfERT-T and TBCRC 022, two studies that involved HER2+ mBC patients with BM [20, 22]. In the NALA trial (NCT01808573), the efficacy and safety of neratinib and capecitabine (N+C) combination therapy was compared against lapatinib plus capecitabine (L+C) in HER2+ mBC patients who had received ≥ 2 prior HER2-directed regimens [23]. Patients in the N+C group was shown to have significantly better progression-free survival [PFS; HR = 0.76 (95% CI 0.63–0.93); *P* = 0.0059] and a lower cumulative incidence of intervention for CNS disease [22.8% (95% CI 15.5–30.9) versus 29.2% (95% CI 22.5–36.1), HR = 0.78; *P* = 0.043] [23]. The most common treatment emergent adverse events (TEAEs) of any grade associated with neratinib in combination with capecitabine were diarrhea, followed by nausea, palmar-plantar erythrodysesthesia syndrome, and vomiting. There was no reported grade 4 diarrhea [23].

Current evidence on the efficacy and safety of neratinib in HER2+ breast cancer has been predominantly derived from the Western population. As previously outlined, ethnic disparities in breast cancer tumor biology exist and may contribute to differing outcomes. Moreover, treatment patterns also vary from region to region. Therefore, a descriptive analysis from the NALA study was performed to establish the efficacy and safety of neratinib in combination with capecitabine in Asian patients with HER2+ mBC, who had received ≥ 2 HER2-directed regimens.

## Patients and methods

### Study design and treatment

NALA is an international, randomized, active-controlled, open-label phase III trial. Eligibility included HER2 overexpression or gene amplification stage IV mBC with ≥ 2 prior HER2-directed regimens. Patients with prior exposure to capecitabine, neratinib, lapatinib, or any other HER2-directed TKIs, and symptomatic or unstable BM were excluded. Patients were randomized 1:1 to receive either N+C or L+C. Randomization was stratified according to the number of previous HER2-directed regimens for mBC (2, or ≥ 3), geographic region (North America, Europe, Rest of world), hormone receptor status (positive vs. negative), and the location of disease (visceral vs. non-visceral only). This subgroup analysis pertains to the Asian patients enrolled from pan-Asian countries, *i.e.*, Hong Kong, Japan, Singapore, South Korea, and Taiwan. The study protocol and amendments were approved by the institutional ethics committee or review board at each participating site. Written informed consent was obtained from all participants.

Patients were randomly assigned to N [240 mg once daily (QD)] + C [750 mg/m^2^ twice daily (BID)] with mandatory loperamide prophylaxis or to L (1250 mg QD) + C (1000 mg/m^2^ BID). Capecitabine was administered on days 1–14 of the 21-day cycle. The study treatment was discontinued when patients developed disease progression or intolerable AE, or received additional/alternative anticancer intervention.

### Outcomes and assessments

The co-primary endpoints were centrally assessed PFS and OS. PFS was assessed per Response Evaluation Criteria in Solid Tumors (RECIST) v1.1 by blinded independent central review. The secondary endpoints included time to intervention for CNS diseases, investigator-assessed PFS, objective response rate (ORR), duration of response (DoR), clinical benefit rate (CBR), safety, and health-related quality of life (HRQoL). Cumulative incidence of progressive CNS disease was also analyzed, based on the available CNS scans. Tumor assessments were performed prior to randomization and at 6-week intervals until disease progression or death. Adverse events were evaluated according to the National Cancer Institute Common Terminology Criteria for Adverse Events version 4.0 and monitored for 28 days following the last dose of the study drug. HRQoL was assessed every 6 weeks using the European Organization for Research and Treatment of Cancer (EORTC) Quality of Life Questionnaire-C30 (QLQ-C30, version 3) until end of treatment.

### Statistical analysis

All patients randomized were included in the ITT population and patients who received at least 1 dose of the study drug were included in the safety analysis. A subgroup analysis was conducted to assess the efficacy and safety of N+C versus L+C in patients enrolled from Asian countries. Time-to-event endpoints were estimated with the Kaplan–Meier method and p values were calculated using the log-rank test. The hazard ratios (HRs) and corresponding 95% confidence intervals (CIs) were calculated using the Cox proportional hazard model. The competing risk model was employed to evaluate time to intervention for CNS disease, with death from any cause as a competing risk. The difference in cumulative incidence between the two treatment arms was tested using the Gray’s Test. Cochran-Mantel–Haenszel Χ^2^ test was used to compare the ORR and CBR between the treatment groups. No adjustments were made for multiplicity. SAS statistical software (version 9.1 or later) was used for all analyses.

## Results

### Patients

Between May 2013 and July 2017, a total of 621 patients were enrolled from 28 countries in the NALA study. Of these, 202 Asian patients (N+C, *n* = 104; L+C, *n* = 98) were enrolled from Asian countries, including Hong Kong, Japan, Singapore, South Korea, and Taiwan. Baseline patient and disease characteristics were fairly comparable between the two treatment arms in this Asian cohort (Table 1). The mean age was 54.8 ± 10.2 years. Nearly three-quarters of the patients had visceral diseases and half of the patients had hormone receptor-negative tumors. Around 70% of the patients had received two HER2-directed regimens prior to randomization.

**Table 1:** Baseline demographics and disease characteristics of the Asian patients

Characteristics	N+C (*n* = 104)	L+C (*n* = 98)	Total (*N* = 202)
Age (years at enrollment)			
Mean (SD)	56.2 (9.9)	53.4 (10.4)	54.8 (10.2)
Age group			
< 65 years	86 (82.7)	84 (85.7)	170 (84.2)
≥ 65 years	18 (17.3)	14 (14.3)	32 (15.8)
Sex			
Female	104 (100)	96 (98.0)	200 (99.0)
Male	0 (0.0)	2 (2.0)	2 (1.0)
ECOG PS at enrollment			
0	69 (66.3)	53 (54.1)	122 (60.4)
1	35 (33.7)	45 (45.9)	80 (39.6)
Hormone receptor status^a^			
Negative	56 (53.8)	48 (49.0)	104 (51.5)
Positive	48 (46.2)	50 (51.0)	98 (48.5)
Disease location			
Non visceral	27 (26.0)	22 (22.4)	49 (24.3)
Visceral	77 (74.0)	76 (77.6)	153 (75.7)
Histological grade at diagnosis			
Well differentiated	4 (3.8)	3 (3.1)	7 (3.5)
Moderately differentiated	34 (32.7)	23 (23.5)	57 (28.2)
Poorly differentiated	41 (39.4)	35 (35.7)	76 (37.6)
Undifferentiated	2 (1.9)	1 (1.0)	3 (1.5)
Unknown	23 (22.1)	36 (36.7)	59 (29.2)
Prior anticancer therapy			
Neoadjuvant	14 (13.5)	18 (18.4)	32 (15.8)
Adjuvant	49 (47.1)	36 (36.7)	85 (42.1)
Metastatic/locally advanced	104 (100.0)	98 (100.0)	202 (100.0)
Number of previous HER2-directed regimens			
2	73 (70.2)	70 (71.4)	143 (70.8)
≥ 3	31 (29.8)	28 (28.6)	59 (29.2)
Prior HER2-directed therapies			
Trastuzumab only	65 (62.5)	56 (57.1)	121 (59.9)
Trastuzumab and pertuzumab	7 (6.7)	10 (10.2)	17 (8.4)
Trastuzumab and T-DM1	14 (13.5)	17 (17.3)	31 (15.3)
Trastuzumab, pertuzumab, and T-DM1	18 (17.3)	15 (15.3)	33 (16.3)
Location of disease at enrollment in the brain			
Yes	18 (17.3)	19 (19.4)	37 (18.3)
No	86 (82.7)	79 (80.6)	165 (81.7)

Data are presented as *n* (%), unless otherwise stated

*ECOG PS* eastern cooperative oncology group performance status, *ER* estrogen receptor, *L*+*C* lapatinib plus capecitabine, *N*+*C* neratinib plus capecitabine, *PR* progesterone receptor, *SD* standard deviation, *T-DM1* trastuzumab emtansine

^a^Hormone receptor positive: ER positive, PR positive, or both. Hormone receptor negative: ER and PR negative

### Efficacy

**Fig. 1:**
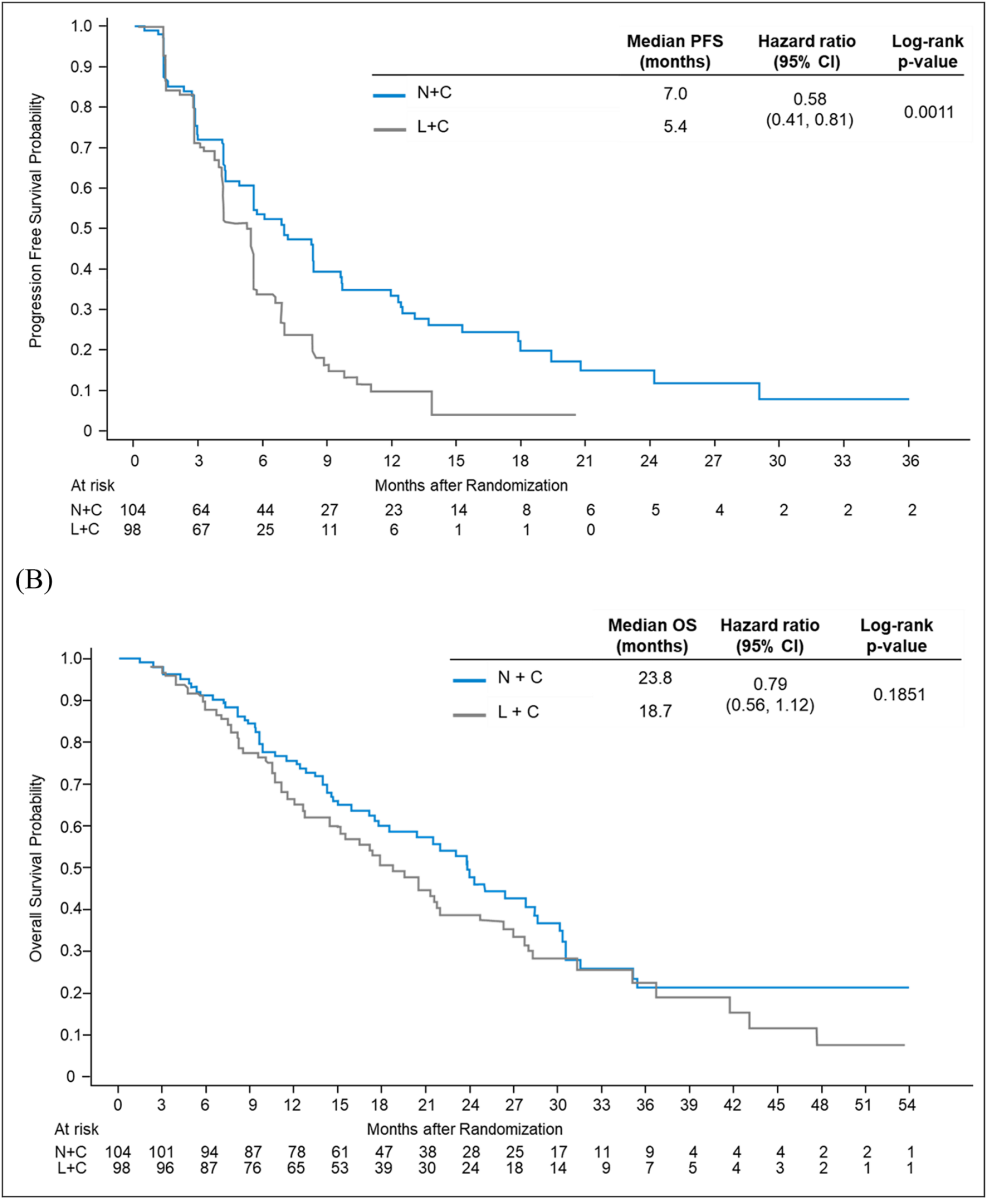
Kaplan–Meier curves for centrally assessed PFS and OS in the Asian subgroup. *CI* confidence interval, *L*+*C* lapatinib plus capecitabine, *N*+*C* neratinib plus capecitabine, *OS* overall survival, *PFS* progression-free survival

Among the Asian mBC patients, the estimated median PFS by central assessment was longer with N+C than that with L+C [7.0 months (95% CI 4.9–8.4) versus 5.4 months (95% CI 4.1–5.6); Log-rank *P* = 0.0011; Fig. 1A]. Kaplan–Meier curves for PFS of N+C and L+C separated at around 24 weeks or 3rd tumor assessment. The PFS benefit of neratinib was consistently seen across most prespecified subgroups although only some had the upper bound of the confidence interval below 1 (Supplementary Figure S1), including age group [< 65 years: HR = 0.58 (95% CI 0.41–0.81)], hormone receptor status [negative: HR = 0.37 (95% CI 0.23–0.60)], disease location [visceral disease: HR = 0.59 (95% CI 0.41–0.86)], and previous HER2 regimens [2 regimens: HR = 0.63 (95% CI 0.43–0.94); ≥ 3 regimens: HR = 0.45 (95% CI 0.23–0.85)]. Median OS was also longer with N+C [23.8 months (95% CI 17.7–28.3) versus 18.7 months (95% CI 14.7–21.9), p = 0.1851; Fig. 1B].

**Fig. 2:**
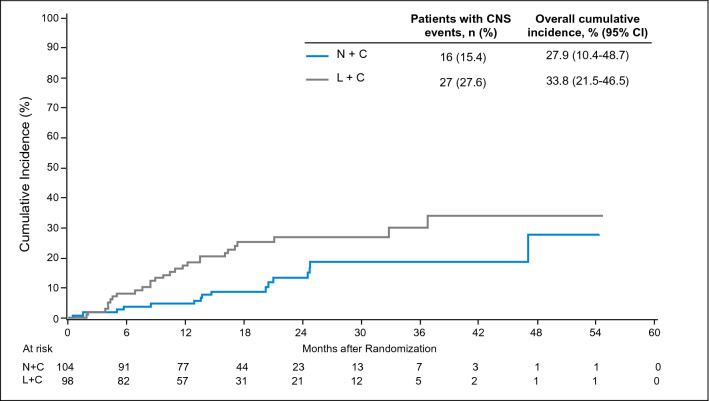
Cumulative incidence of intervention for CNS disease in the Asian cohort. *CI* confidence interval, *L*+*C* lapatinib plus capecitabine, *N*+*C* neratinib plus capecitabine

Of the 202 Asian patients, 43 patients had interventions for CNS disease. Sixteen (15.4%) and 27 (27.6%) patients in the N+C and L+C group had interventions for CNS disease, respectively. The overall cumulative incidence of intervention for CNS disease was lower for the N+C group than for the L+C group [27.9%, (95% CI 10.4–48.7) versus 33.8% (95% CI 21.5–46.5); Gray’s test for equality *P* = 0.039] (Fig. 2), and a considerable difference between the two arms were noted in the first 18 month (Table 2).

**Table 2:** Summary of efficacy endpoints findings in the Asian cohort

Variable	N+C (*n* = 104)	L+C (*n* = 98)	HR (95% CI)	*P* value
PFS^a^				
Median, months (95% CI)	7.0 (4.9–8.4)	5.4 (4.1–5.6)	0.58^b^ (0.41–0.81)	0.0011^¥^
Kaplan–Meier estimate, % (95% CI)				
6 months	53.7 (42.9–63.3)	33.8 (23.9–43.8)	–	–
12 months	33.5 (23.4–43.9)	10.0 (4.4–18.6)	–	–
18 months	19.9 (11.2–30.4)	4.0 (0.8–11.6)	–	–
OS				
Median, months (95% CI)	23.8 (17.7–28.3)	18.7 (14.7–21.9)	0.79^b^ (0.56–1.12)	0.1851^¥^
Kaplan–Meier estimate, % (95% CI)				
12 months	75.7 (66.2–82.9)	66.3 (56.1–74.7)	–	–
18 months	60.0 (49.6–68.9)	50.5 (39.9–60.2)	–	–
24 months	47.7 (36.6–58.1)	38.9 (28.4–49.3)	–	–
Cumulative incidence estimate of intervention for CNS disease, % (95% CI)				
6 months	3.9 (1.3–8.9)	8.2 (3.8–14.7)	–	–
12 months	4.9 (1.8–10.2)	17.4 (10.6–25.5)	–	–
18 months	8.8 (4.3–15.3)	25.2 (16.9–34.4)	–	–
Overall	27.9 (10.4–48.7)	33.8 (21.5–46.5)	–	0.039^*^
Best overall response, n (%)	N+C (*N* = 81)	L+C (*N* = 84)		
CR	4 (4.9)	1 (1.2)	–	–
PR	39 (48.1)	30 (35.7)	–	–
SD	24 (29.6)	39 (46.4)	–	–
PD	12 (14.8)	13 (15.5)	–	–
Unavailable	2 (2.5)	1 (1.2)	–	–
ORR, n (%)^c^	33 (40.7)	27 (32.1)	–	0.3388^ǂ^
95% CI	29.9–52.2	22.4–43.2		
Median DoR, months (95% CI)	11.1 (6.9–22.9)	4.2 (4.1–5.6)		
CBR, n (%)^c^	42 (51.9)	34 (40.5)	–	0.1699^ǂ^
95% CI	40.5–63.1	29.9–51.7		

*CBR* clinical benefit rate, *CI* confidence interval, *CNS* central nervous system, *CR* complete response, *DoR* duration of response, *HR* hazard ratio, *L*+*C* lapatinib plus capecitabine, *mo* month, *N*+*C* neratinib plus capecitabine, *ORR* objective response rate, *OS* overall survival, *PD* disease progression, *PFS* progression-free survival, *PR* partial response, *SD* stable disease

^a^End point was evaluated by the independent review committee

^b^Cox proportional hazards model

^**c**^Confirmed responses in patients with measurable disease (N+C: *n* = 81; L+C: *n* = 84)

^¥^The *P* value was calculated with the 2-sided log-rank test

^*^Gray’s Test for Equality of CNS Cumulative incidence

^ǂ^The *P* value was calculated with Cochran–Mantel–Haenszel test

Among patients with measurable disease at baseline (n = 165), ORR was 40.7% (95% CI 29.9–52.2) in the N+C and 32.1% (95% CI 22.4–43.2) in the L+C group (Table 2). CBR was 51.9% (95% CI 40.5–63.1) and 40.5% (95% CI 29.9–51.7) in the N+C and L+C group, respectively (Table 2). Median DoR was longer with N+C, compared with L+C [11.1 months (95% CI 6.9–22.9) versus 4.2 months (95% CI 4.1–5.6); *P* < 0.0001]. A considerably larger proportion of patients had responses that lasted ≥ 12 months with neratinib (45.6% versus 4.3%).

### Safety and HRQoL

The median treatment duration of neratinib and lapatinib was 6.6 and 5.2 months, respectively. While dose reductions and dose holds were more frequently performed in neratinib-treated patients, a greater proportion of patients treated with neratinib received ≥ 12 month treatments (26.0% versus 8.2%) (Supplementary Table S1). No new safety signals were observed in this Asian cohort. All patients experienced TEAEs of any grade. The incidence of grade 3 TEAE was slightly higher in the N+C group than in the L+C group (53.8% versus 48.0%). Serious adverse event (SAE) was reported in 31.7% and 36.7% of the patients in the N+C and L+C group, respectively.

The incidence of TEAE leading to hospitalization and treatment discontinuation was similar between the two treatment groups. The number and incidence of TEAE-related hospitalization were 29.8% (*n* = 31) and 34.7% (*n* = 34) in the N+C and L+C group, respectively. TEAE led to treatment discontinuation in 11.5% (*n* = 12) and 14.3% (n = 14) of the patients in the N+C and L+C group, respectively. Seven (6.7%) and 5 (5.1%) patients, respectively, underwent neratinib and lapatinib dose reduction due to TEAE.

The most frequently reported TEAEs of any grade in the Asian subgroup were diarrhea, palmar-plantar erythrodysaesthesia (PPE) syndrome, and vomiting (Table 3). Grade 3 diarrhea occurred more frequently in patients treated with neratinib than with lapatinib (25.0% versus 6.1%) and was concentrated during the first cycle (Supplementary Figure S2). No grade 4 diarrhea was documented. Dose reduction due to diarrhea was required for 4 patients (3.8%) and 1 patient (1.0%) in the N+C and L+C arm, respectively. Diarrhea leading to permanent treatment discontinuation was reported in 1 patient in the N+C group.

**Table 3:** TEAEs reported in ≥ 10% of Asian patients in the safety population

AE—*n* (%)	N+C (*n* = 104)		L+C (*n* = 98)	
	All grade	Grade 3/4	All grade	Grade 3/4
Diarrhea	82 (78.8)	26 (25.0)	50 (51.0)	6 (6.1)
Palmar-plantar erythrodysaesthesia	53(51.0)	12 (11.5)	67 (68.4)	9 (9.2)
Vomiting	49 (47.1)	4 (3.8)	21 (21.4)	0 (0.0)
Decreased appetite	42 (40.4)	2 (1.9)	18 (18.4)	3 (3.1)
Nausea	42 (40.4)	1 (1.0)	28 (28.6)	1 (1.0)
Fatigue	29 (27.9)	0 (0.0)	24 (24.5)	1 (1.0)
Constipation	25 (24.0)	1 (1.0)	11 (11.2)	1 (1.0)
Weight decreased	25 (24.0)	1 (1.0)	14 (14.3)	1 (1.0)
Stomatitis	23 (22.1)	2 (1.9)	28 (28.6)	4 (4.1)
Paronychia	17 (16.3)	2 (1.9)	25 (25.5)	1 (1.0)
Dizziness	15 (14.4)	0 (0.0)	14 (14.3)	1 (1.0)
Cough	14 (13.5)	0 (0.0)	13 (13.3)	0 (0.0)
Anemia	13 (12.5)	2 (1.9)	16 (16.3)	5 (5.1)
Pruritus	13 (12.5)	0 (0.0)	11 (11.2)	0 (0.0)
Upper respiratory tract infection	13 (12.5)	0 (0.0)	7(7.1)	0 (0.0)
Abdominal distension	11 (10.6)	0 (0.0)	6 (6.1)	2 (2.0)
Pyrexia	11 (10.6)	0 (0.0)	10 (10.2)	1 (1.0)
Headache	10 (9.6)	1 (1.0)	14 (14.3)	3 (3.1)
Rash	9 (8.7)	0 (0.0)	22 (22.4)	2 (2.0)
Hypokalaemia	8 (7.7)	3 (2.9)	13 (13.3)	6 (6.1)
Dermatitis acneiform	7 (6.7)	0 (0.0)	11 (11.2)	0 (0.0)

AEs were graded per the National Cancer Institute Common Terminology Criteria for Adverse Events version 4.0

A treatment emergent adverse event (TEAE) was defined as any AE that occurred or worsened on or after the first dose of study drug and up to 28 days following the last dose

*AEs* adverse events, *L*+*C* lapatinib+capecitabine, *N*+*C* neratinib+capecitabine

Clinically significant adverse cardiac events were scarce in both treatment arms. One case of pericardial effusion was reported in each of the treatment arm. A case of cardiac tamponade occurred in the N+C group, and one case of acute myocardial infarction had been reported in the L+C group.

A total of 198 patients completed the EORTC QLQ-C30, yielding a questionnaire completion rate of 98%. The mean EORTC QLQ-C30-Global Health Status scores were comparable between the two arms throughout the treatment period (Supplementary Figure S3).

## Discussion

A persistent rise in breast cancer incidence has been perceived in Asia throughout past decades [24, 25]. While a gradual decline in breast cancer-related mortality has been noted in the western world in recent decades, a steady increase has been reported in numerous Asian countries [26]. Aside from epidemiological differences, disparities in tumor characteristics, pharmacogenomics, and access to treatments also exist between Asian breast cancer patients and their western counterparts, all of which may have contributed to the differing outcomes between the two populations [4, 27]. To build up the evidence of neratinib in HER2+ Asian mBC patients, this descriptive analysis was performed on the NALA trial, which involved 202 Asian patients.

In this analysis of pan-Asian subgroup of patients with HER2+ mBC, neratinib in combination with capecitabine was associated with longer median PFS and DoR, compared with lapatinib and capecitabine. Patients in the neratinib arm also had fewer interventions for CNS disease, implying that neratinib combination therapy may delay CNS progression. Albeit not statistically significant, a trend for longer OS was also seen in patients receiving N+C. ORR and CBR were both higher for the N+C arm in comparison to the L+C arm. These efficacy results were consistent with those reported by Saura et al. in the main study [23]. In the Asian subgroup, the most frequently reported TEAEs of any grade in both arms were diarrhea, PPE syndrome, followed by vomiting (Table 3). Similarly, diarrhea (N+C: 83.2%; L+C: 66.2%), nausea (N+C: 53.1%; L+C: 42.4%), PPE syndrome (N+C: 45.9%; L+C: 56.3%) and vomiting (N+C: 45.5%; L+C: 31.2%) were the most common TEAEs in the overall population [23]. While the incidence and severity of the most frequent TEAEs were fairly comparable between the Asian subgroup and the overall population, nausea was found to be moderately less often reported among the Asian patients (N+C: 40.4% versus 53.1%; L+C: 28.6% versus 42.4%) [23]. Compared to the overall population who received N+C, the incidence of diarrhea-related dose reduction (3.8% versus 5.3%) and treatment discontinuation (1.0% versus 2.6%) was also lower in the Asian subgroup [23].

Compared with the overall study population, a slightly larger effect size was noted in this Asian cohort, in terms of PFS and DoR. Factors that may have contributed to this finding are complex and multifactorial, as formerly described in the literatures [4, 5]. In this Asian cohort, we noted a greater proportion of patients aged < 65 years and had Eastern Cooperative Oncology Group (ECOG) performance status of 0, compared with the overall study population. This subgroup also had a higher proportion of hormone receptor-negative tumors, which is a feature that has been associated with greater benefit from N+C [23, 28]. Similar to real-world practice, the proportion of patients who have had trastuzumab-only treatments in the mBC setting was higher among the Asians enrolled from pan-Asian countries, which may also have likely affected treatment outcomes. In addition, the Asians had a higher exposure to the study drugs than the overall population, as the latter had a shorter median treatment duration and a higher discontinuation rate due to TEAEs. Nevertheless, whether the difference in PFS and DoR may confer to a more favorable OS outcome warrants a longer follow-up.

The incidence of BM has notably increased among HER2+ breast cancer patients since the introduction of trastuzumab [29]. While continued trastuzumab treatments among patients with BM have been associated with significant OS benefits, as opposed to non-trastuzumab-based regimens [30, 31], agents with better CNS penetration have been sought. However, the accrual of clinical evidence on CNS activity or efficacy has been heavily hampered, as presence of BM often precludes patients from trial entry. Lapatinib is one of the first HER2-targeted agents that has demonstrated potential in reducing the risk of BM development and progression. Compared with capecitabine monotherapy, the addition of lapatinib showed promise in reducing the risk of BM development in HER2+ locally advanced or mBC patients progressing after systemic treatments such as anthracycline, taxane, and trastuzumab in early phase II and III studies [32, 33]. In the subsequent LANDSCAPE trial, treatment with L+C yielded a CNS ORR of 65.9% among patients with BM without prior whole brain radiation therapy [34]. Albeit seemingly encouraging, these observations were gleaned from small patient numbers. While trial data on the intracranial efficacy of other HER2-directed agents remain meager, favorable preliminary results have been reported in KAMILLA of ado-trastuzumab emtansine (T-DM1) monotherapy [35], DESTINY-Breast01 of trastuzumab deruxtecan (DS-8201) [36], and HER2CLIMB of tucatinib+capecitabine+trastuzumab combination therapy [37]. In particular, among locally advanced or metastatic HER2+ patients with BM who had received trastuzumab, pertuzumab, and T-DM1, the combination of tucatinib with capecitabine and trastuzumab has been shown to not only significantly reduce the risk of intracranial progression or death by 68%, but also prolonged median OS by 6 months, as compared with capecitabine+trastuzumab [38].

Neratinib, on the other hand, has a relatively more established efficacy in the CNS when combined with chemotherapy. As a first-line HER2-directed treatment in patients with advanced breast cancer, the combination of neratinib and paclitaxel has been shown to lower the risk of CNS recurrence by 52%, compared with trastuzumab+paclitaxel [22]. In HER2+ mBC patients with BM, N+C was associated with a CNS ORR of 49% and 33% in lapatinib-naïve and lapatinib-pretreated patients, respectively [39]. Lastly, among a more heavily treated HER2+ mBC population, a reduced cumulative incidence of intervention for CNS disease was still seen with N+C, as compared with L+C (22.8% vs. 29.2%) [23].

Diarrhea is the most commonly reported toxicity with neratinib, which most frequently occurred during the first cycle. With mandatory loperamide prophylaxis, the incidence of grade 3 diarrhea was reduced to about 25% in both the Asian and overall population in the NALA study [23]. Furthermore, diarrhea did not seem to significantly impact patients’ quality of life, according to the HRQoL results. Alternatively, neratinib tolerability may be further improved with strategies including preemptive prophylaxis with loperamide+budesonide, loperamide+colestipol, and the incorporation of a step-wise dose escalation to the starting dose [40].

While this study provides relevant insights into the efficacy and safety of neratinib in the Asian population, some limitations are acknowledged. As with most descriptive analyses, our analysis does not necessarily have sufficient power to facilitate stringent comparisons between the two treatment arms. Although geographic region was one of the randomization stratification factors, the prespecified subgroups included only North America, Europe, and rest of world. Hence, patient and disease characteristics in the Asian cohort were not as well-balanced as that in the overall population. There was a slightly higher proportion of hormone receptor-negative and poorly differentiated tumors in the N+C arm. Also, a greater proportion of patients in the N+C group had previously received trastuzumab-only treatments.

In summary, combination therapy of neratinib and capecitabine was associated with prolonged PFS, DoR, and the time to intervention for CNS disease among HER2+ Asian patients with mBC who had previously received ≥ 2 HER2-directed regimens. The efficacy and safety profiles of N+C in the Asian cohort were consistent with those in the overall population. For HER2+ mBC patients who received trastuzumab-only regimens for their metastatic disease, neratinib may offer additional benefits.

## Supplementary Information

Below is the link to the electronic supplementary material.

Supplementary file1 (PPTX 62 kb)

Supplementary file2 (DOCX 14 kb)

## Data Availability

The datasets generated during and/or analyzed during the current study are available from the corresponding author on reasonable request.
