# Supplementary material for: Analysis of the pan-Asian subgroup of patients in the NALA Trial: a randomized phase III NALA Trial comparing neratinib+capecitabine (N+C) vs lapatinib+capecitabine (L+C) in patients with HER2+metastatic breast cancer (mBC) previously treated with two or more HER2-directed regimens
Source: Breast Cancer Res Treat. 2021 Sep 23;189(3):665–76. doi: 10.1007/s10549-021-06313-5 (PMC8505315; doi:10.1007/s10549-021-06313-5)
Supplement: Supplementary file 1 — Supplementary file1 (PPTX 62 kb) [file 10549_2021_6313_MOESM1_ESM.pptx]

## Slide 1
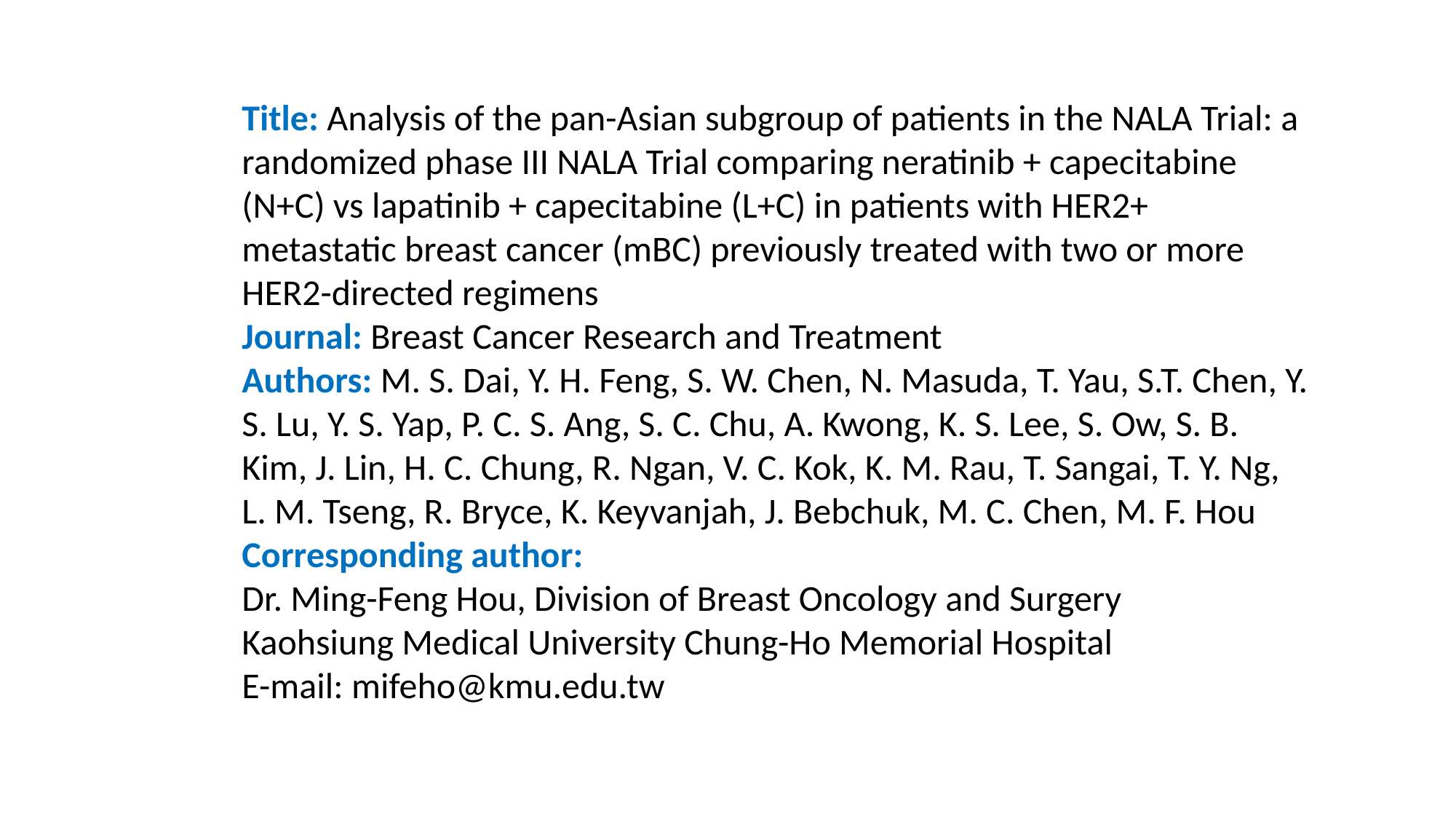

Title: Analysis of the pan-Asian subgroup of patients in the NALA Trial: a randomized phase III NALA Trial comparing neratinib + capecitabine (N+C) vs lapatinib + capecitabine (L+C) in patients with HER2+ metastatic breast cancer (mBC) previously treated with two or more HER2-directed regimens
Journal: Breast Cancer Research and Treatment
Authors: M. S. Dai, Y. H. Feng, S. W. Chen, N. Masuda, T. Yau, S.T. Chen, Y. S. Lu, Y. S. Yap, P. C. S. Ang, S. C. Chu, A. Kwong, K. S. Lee, S. Ow, S. B. Kim, J. Lin, H. C. Chung, R. Ngan, V. C. Kok, K. M. Rau, T. Sangai, T. Y. Ng, L. M. Tseng, R. Bryce, K. Keyvanjah, J. Bebchuk, M. C. Chen, M. F. Hou
Corresponding author:
Dr. Ming-Feng Hou, Division of Breast Oncology and Surgery
Kaohsiung Medical University Chung-Ho Memorial Hospital
E-mail: mifeho@kmu.edu.tw

## Slide 2
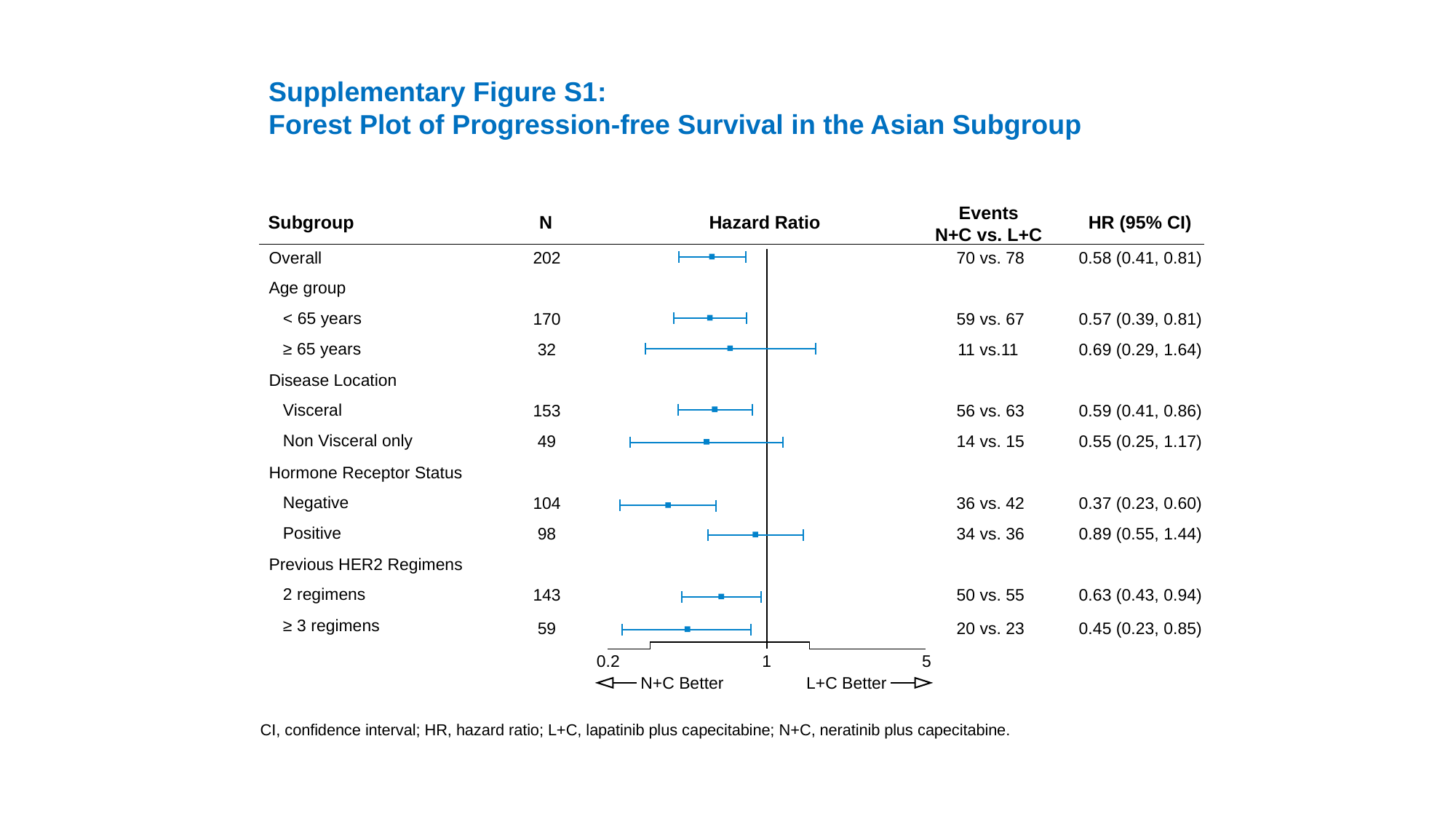

Supplementary Figure S1:
Forest Plot of Progression-free Survival in the Asian Subgroup
Events
N+C vs. L+C
Subgroup
N
Hazard Ratio
HR (95% CI)
| Overall | 202 | | 70 vs. 78 | 0.58 (0.41, 0.81) |
| --- | --- | --- | --- | --- |
| Age group | | | | |
| < 65 years | 170 | | 59 vs. 67 | 0.57 (0.39, 0.81) |
| ≥ 65 years | 32 | | 11 vs.11 | 0.69 (0.29, 1.64) |
| Disease Location | | | | |
| Visceral | 153 | | 56 vs. 63 | 0.59 (0.41, 0.86) |
| Non Visceral only | 49 | | 14 vs. 15 | 0.55 (0.25, 1.17) |
| Hormone Receptor Status | | | | |
| Negative | 104 | | 36 vs. 42 | 0.37 (0.23, 0.60) |
| Positive | 98 | | 34 vs. 36 | 0.89 (0.55, 1.44) |
| Previous HER2 Regimens | | | | |
| 2 regimens | 143 | | 50 vs. 55 | 0.63 (0.43, 0.94) |
| ≥ 3 regimens | 59 | | 20 vs. 23 | 0.45 (0.23, 0.85) |
0.2
1
5
N+C Better
L+C Better
CI, confidence interval; HR, hazard ratio; L+C, lapatinib plus capecitabine; N+C, neratinib plus capecitabine.

## Slide 3
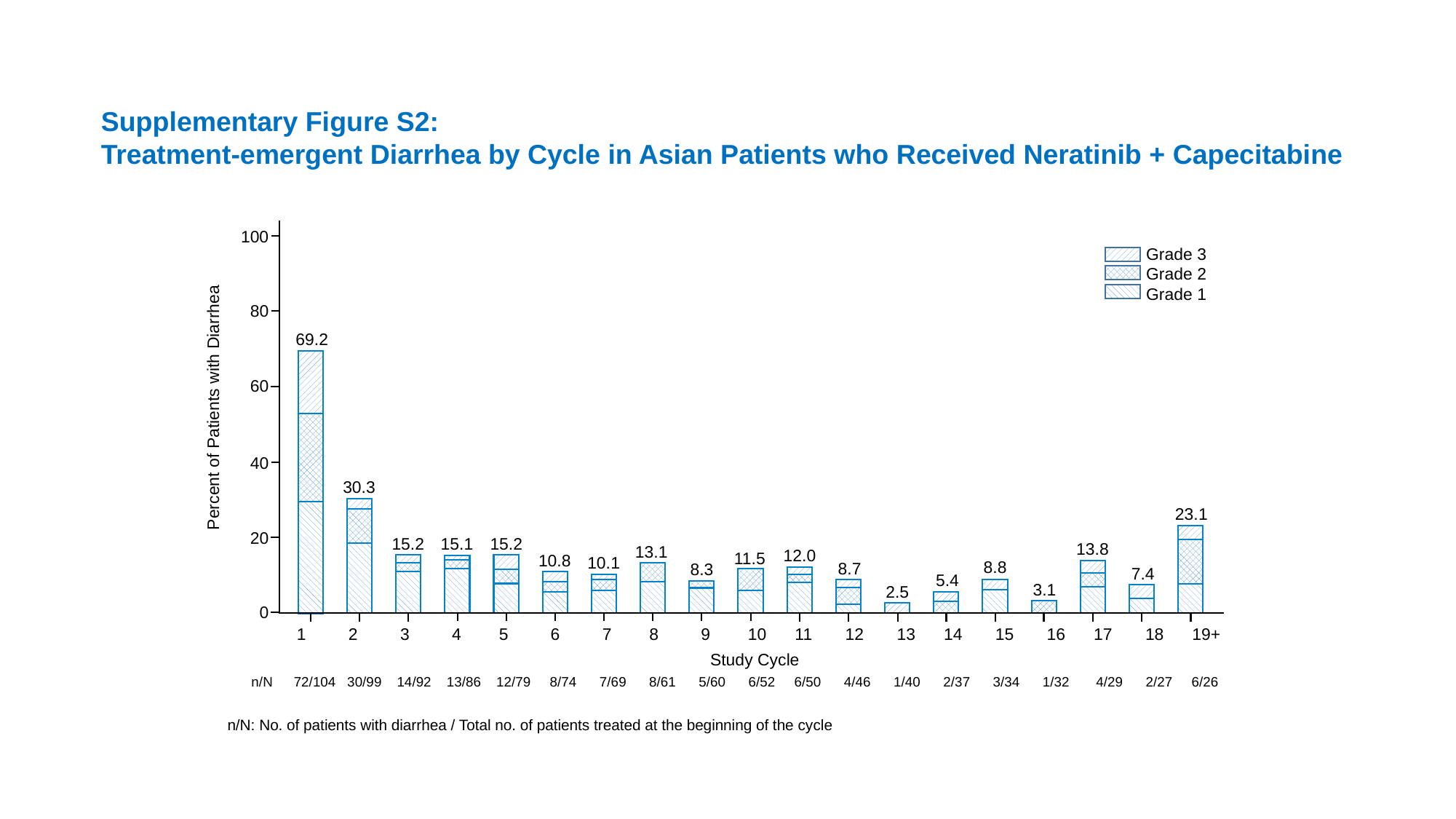

Supplementary Figure S2:
Treatment-emergent Diarrhea by Cycle in Asian Patients who Received Neratinib + Capecitabine
100
80
60
40
20
0
Grade 3
Grade 2
Grade 1
Percent of Patients with Diarrhea
69.2
30.3
23.1
15.1
15.2
15.2
13.8
13.1
12.0
11.5
10.8
10.1
8.8
8.7
8.3
7.4
5.4
3.1
2.5
1 2 3 4 5 6 7 8 9 10 11 12 13 14 15 16 17 18 19+
Study Cycle
n/N
 72/104 30/99 14/92 13/86 12/79 8/74 7/69 8/61 5/60 6/52 6/50 4/46 1/40 2/37 3/34 1/32 4/29 2/27 6/26
n/N: No. of patients with diarrhea / Total no. of patients treated at the beginning of the cycle

## Slide 4
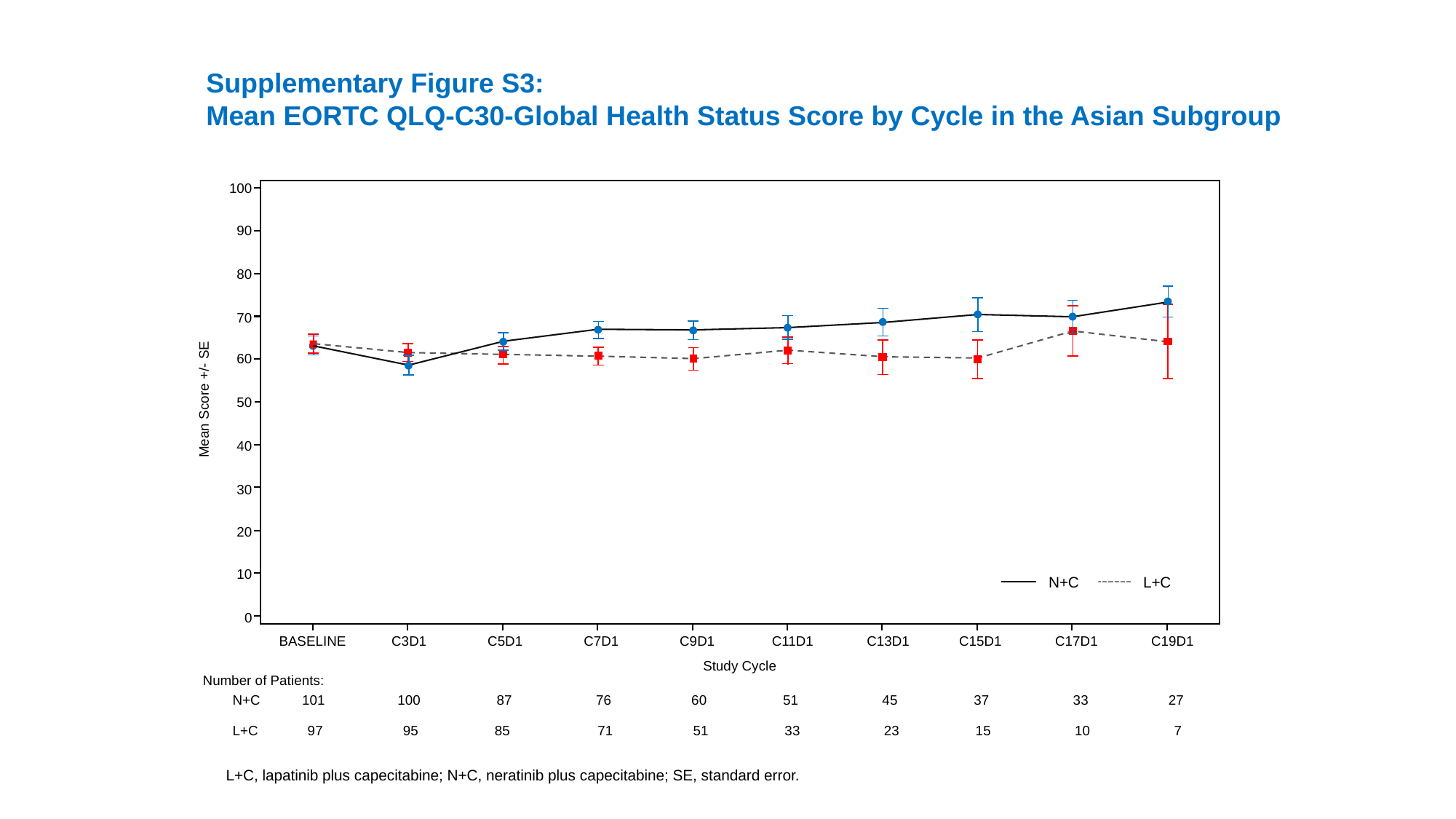

Supplementary Figure S3:
Mean EORTC QLQ-C30-Global Health Status Score by Cycle in the Asian Subgroup
100
90
80
70
60
50
40
30
20
10
 0
BASELINE C3D1 C5D1 C7D1 C9D1 C11D1 C13D1 C15D1 C17D1 C19D1
Mean Score +/- SE
N+C
L+C
Study Cycle
Number of Patients:
N+C 101 100 87 76 60 51 45 37 33 27
L+C 97 95 85 71 51 33 23 15 10 7
L+C, lapatinib plus capecitabine; N+C, neratinib plus capecitabine; SE, standard error.
