# Supplementary material for: Analysis of the pan-Asian subgroup of patients in the NALA Trial: a randomized phase III NALA Trial comparing neratinib+capecitabine (N+C) vs lapatinib+capecitabine (L+C) in patients with HER2+metastatic breast cancer (mBC) previously treated with two or more HER2-directed regimens
Source: Breast Cancer Res Treat. 2021 Sep 23;189(3):665–76. doi: 10.1007/s10549-021-06313-5 (PMC8505315; doi:10.1007/s10549-021-06313-5)
Supplement: Supplementary file 2 — Supplementary file2 (DOCX 14 kb) [file 10549_2021_6313_MOESM2_ESM.docx]

**Title:**

**Analysis of the pan-Asian subgroup of patients in the NALA Trial: a randomized phase III NALA Trial comparing neratinib + capecitabine (N+C) vs lapatinib + capecitabine (L+C) in patients with HER2+ metastatic breast cancer (mBC) previously treated with two or more HER2-directed regimens**

**Journal: Breast Cancer Research and Treatment**

**Authors:**

**M. S. Dai, Y. H. Feng, S. W. Chen, N. Masuda, T. Yau, S.T. Chen, Y. S. Lu, Y. S. Yap, P. C. S. Ang, S. C. Chu, A. Kwong, K. S. Lee, S. Ow, S. B. Kim, J. Lin, H. C. Chung, R. Ngan, V. C. Kok, K. M. Rau, T. Sangai, T. Y. Ng, L. M. Tseng, R. Bryce, K. Keyvanjah, J. Bebchuk, M. C. Chen, M. F. Hou**

**Corresponding author:**

**Dr. Ming-Feng Hou, Division of Breast Oncology and Surgery**

**Kaohsiung Medical University Chung-Ho Memorial Hospital**

**E-mail: mifeho@kmu.edu.tw**

**Supplementary Table S1: Summary of Neratinib/Lapatinib Exposure in the Asian Subgroup**

|  | **N+C**  **(*n*=104)** | **L+C**  **(*n*=98)** | **Total**  **(*n*=202)** |
| --- | --- | --- | --- |
| **Duration of treatment, n (%)** | | | |
| Median (mo) | 6.55 | 5.19 | 5.52 |
| >=0 to < 3 mo | 27 (26.0) | 28 (28.6) | 55 (27.2) |
| >=3 to < 6 mo | 24 (23.1) | 38 (38.8) | 62 (30.7) |
| >=6 to < 12 mo | 26 (25.0) | 24 (24.5) | 50 (24.8) |
| >=12 mo | 27 (26.0) | 8 (8.2) | 35 (17.3) |
| **Dose reduction, n (%)** | | | |
|  | 23 (22.1) | 14 (14.3) | 37 (18.3) |
| **Dose hold, n (%)** | | | |
|  | 43 (41.3) | 34 (34.7) | 77 (38.1) |

L+C, lapatinib plus capecitabine; mo, month; N+C, neratinib plus capecitabine.
